# Supplementary material for: Surface Microbiome Profiling of Dental Elevators Using Third‐Generation Sequencing: Implications for Infection Control in Dental Practice
Source: Microbiologyopen. 2025 Dec 8;14(6):e70178. doi: 10.1002/mbo3.70178 (PMC12685768; doi:10.1002/mbo3.70178)
Supplement: Supplementary file 1 — customer_backup. [file MBO3-14-e70178-s001.zip › customer_backup/customer_backup/taxa_summary/heatmap/treat/treat.phylum.reabundance.heatmap.pdf]

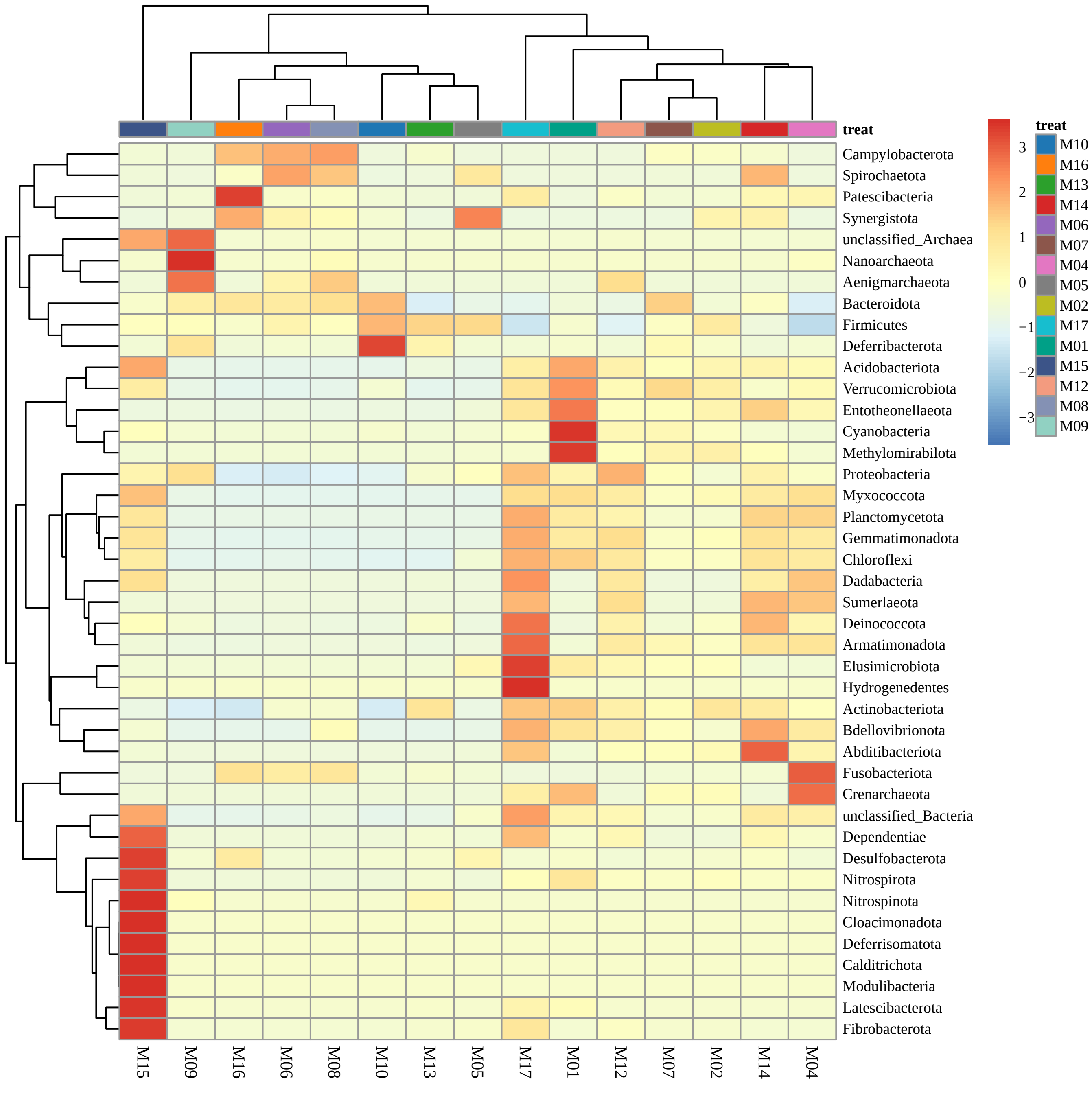

treat
Campylobacterota
Spirochaetota
Patescibacteria
Synergistota
unclassified_Archaea
Nanoarchaeota
Aenigmarchaeota
Bacteroidota
Firmicutes
Deferribacterota
Acidobacteriota
Verrucomicrobiota
Entotheonellaeota
Cyanobacteria
Methylomirabilota
Proteobacteria
Myxococcota
Planctomycetota
Gemmatimonadota
Chloroflexi
Dadabacteria
Sumerlaeota
Deinococcota
Armatimonadota
Elusimicrobiota
Hydrogenedentes
Actinobacteriota
Bdellovibrionota
Abditibacteriota
Fusobacteriota
Crenarchaeota
unclassified_Bacteria
Dependentiae
Desulfobacterota
Nitrospirota
Nitrospinota
Cloacimonadota
Deferrisomatota
Calditrichota
Modulibacteria
Latescibacterota
Fibrobacterota
M15
M09
M16
M06
M08
M10
M13
M05
M17
M01
M12
M07
M02
M14
M04
3
2
1
0
−1
−2
−3
treat
M10
M16
M13
M14
M06
M07
M04
M05
M02
M17
M01
M15
M12
M08
M09
